# Author Correction: Corruption of the Pearson correlation coefficient by measurement error and its estimation, bias, and correction under different error models

**DOI:** 10.1038/s41598-023-46128-6

**Published:** 2023-12-20

**Authors:** Edoardo Saccenti, Margriet H. W. B. Hendriks, Age K. Smilde

**Affiliations:** 1 Laboratory of Systems and Synthetic Biology, Wageningen University & Research, Wageningen, The Netherlands; 2 DSM Biotechnology Center, Delft, The Netherlands; 3 Biosystems Data Analysis, Swammerdam Institute for Life Sciences, University of Amsterdam, Amsterdam, The Netherlands

Correction to: *Scientific Reports* https://doi.org/10.1038/s41598-019-57247-4, published online 16 January 2020

This Article contains errors in the Equations (39), (41), (48), and (78).

**Equation 39:**

39 $$ A^{m} = \frac{1}{{\sqrt {1 + \left( {1 + \frac{{\mu_{{x_{0} }}^{2} }}{{\sigma_{{x_{0} }}^{2} }}} \right)\left( {\frac{{\sigma_{{mu_{x} }}^{2} }}{{\sigma_{{x_{0} }}^{2} }} + \frac{{\sigma_{mc}^{2} }}{{\sigma_{{x_{0} }}^{2} }}} \right)} \sqrt {1 + \left( {1 + \frac{{\mu_{{y_{0} }}^{2} }}{{\sigma_{{y_{0} }}^{2} }}} \right)\left( {\frac{{\sigma_{{mu_{y} }}^{2} }}{{\sigma_{{y_{0} }}^{2} }} + \frac{{\sigma_{mc}^{2} }}{{\sigma_{{y_{0} }}^{2} }}} \right)} }}. $$

should read:

39 $$ A^{m} = \frac{1}{{\sqrt {1 + \left( {1 + \frac{{\mu_{{x_{0} }}^{2} }}{{\sigma_{{x_{0} }}^{2} }}} \right)\left( {\sigma_{{mu_{x} }}^{2} + \sigma_{mc}^{2} } \right)} \times \sqrt {1 + \left( {1 + \frac{{\mu_{{y_{0} }}^{2} }}{{\sigma_{{y_{0} }}^{2} }}} \right)\left( {\sigma_{{mu_{y} }}^{2} + \sigma_{mc}^{2} } \right)} }} $$

**Equation 41:**

41 $$ A^{r} = \frac{1}{{\sqrt {1 + \left( {1 + \frac{{\mu_{{x_{0} }}^{2} }}{{\sigma_{{x_{0} }}^{2} }}} \right)\left( {\frac{{\sigma_{{mu_{x} }}^{2} }}{{\sigma_{{x_{0} }}^{2} }} + \frac{{\sigma_{mc}^{2} }}{{\sigma_{{x_{0} }}^{2} }}} \right) + \frac{{\sigma_{{au_{x} }}^{2} }}{{\sigma_{{x_{0} }}^{2} }} + \frac{{\sigma_{ac}^{2} }}{{\sigma_{{x_{0} }}^{2} }}} \sqrt {1 + \left( {1 + \frac{{\mu_{{y_{0} }}^{2} }}{{\sigma_{{y_{0} }}^{2} }}} \right)\left( {\frac{{\sigma_{{mu_{y} }}^{2} }}{{\sigma_{{y_{0} }}^{2} }} + \frac{{\sigma_{mc}^{2} }}{{\sigma_{{y_{0} }}^{2} }}} \right) + \frac{{\sigma_{{au_{y} }}^{2} }}{{\sigma_{{y_{0} }}^{2} }} + \frac{{\sigma_{ac}^{2} }}{{\sigma_{{y_{0} }}^{2} }}} }}. $$

should read:

41 $$ A^{r} = \frac{1}{{\sqrt {1 + \left( {1 + \frac{{\mu_{{x_{0} }}^{2} }}{{\sigma_{{x_{0} }}^{2} }}} \right)\left( {\sigma_{{mu_{x} }}^{2} + \sigma_{mc}^{2} } \right) + \frac{{\sigma_{{au_{x} }}^{2} }}{{\sigma_{{x_{0} }}^{2} }} + \frac{{\sigma_{ac}^{2} }}{{\sigma_{{x_{0} }}^{2} }}} \times \sqrt {1 + \left( {1 + \frac{{\mu_{{y_{0} }}^{2} }}{{\sigma_{{y_{0} }}^{2} }}} \right)\left( {\sigma_{{mu_{y} }}^{2} + \sigma_{mc}^{2} } \right) + \frac{{\sigma_{{au_{y} }}^{2} }}{{\sigma_{{y_{0} }}^{2} }} + \frac{{\sigma_{ac}^{2} }}{{\sigma_{{y_{0} }}^{2} }}} }}. $$

**Equation 48:**

48 $$ A^{r} = \frac{1}{{\sqrt {1 + \left( {1 + \frac{{\mu_{{x_{0} }}^{2} }}{{\sigma_{{x_{0} }}^{2} }}} \right)\left( {\frac{{\sigma_{{mu_{x} }}^{2} }}{{\sigma_{{x_{0} }}^{2} }} + \frac{{\sigma_{{mc_{x} }}^{2} }}{{\sigma_{{x_{0} }}^{2} }}} \right) + \frac{{\sigma_{{au_{x} }}^{2} }}{{\sigma_{{x_{0} }}^{2} }} + \frac{{\sigma_{{ac_{x} }}^{2} }}{{\sigma_{{x_{0} }}^{2} }}} \times \sqrt {1 + \left( {1 + \frac{{\mu_{{y_{0} }}^{2} }}{{\sigma_{{y_{0} }}^{2} }}} \right)\left( {\frac{{\sigma_{{mu_{y} }}^{2} }}{{\sigma_{{y_{0} }}^{2} }} + \frac{{\sigma_{{mc_{y} }}^{2} }}{{\sigma_{{y_{0} }}^{2} }}} \right) + \frac{{\sigma_{{au_{y} }}^{2} }}{{\sigma_{{y_{0} }}^{2} }} + \frac{{\sigma_{{ac_{y} }}^{2} }}{{\sigma_{{y_{0} }}^{2} }}} }}. $$

should read:

48 $$ A^{r} = \frac{1}{{\sqrt {1 + \left( {1 + \frac{{\mu_{{x_{0} }}^{2} }}{{\sigma_{{x_{0} }}^{2} }}} \right)\left( {\sigma_{{mu_{x} }}^{2} + \sigma_{{mc_{x} }}^{2} } \right) + \frac{{\sigma_{{au_{x} }}^{2} }}{{\sigma_{{x_{0} }}^{2} }} + \frac{{\sigma_{{ac_{x} }}^{2} }}{{\sigma_{{x_{0} }}^{2} }}} \times \sqrt {1 + \left( {1 + \frac{{\mu_{{y_{0} }}^{2} }}{{\sigma_{{y_{0} }}^{2} }}} \right)\left( {\sigma_{{mu_{y} }}^{2} + \sigma_{{mc_{y} }}^{2} } \right) + \frac{{\sigma_{{au_{y} }}^{2} }}{{\sigma_{{y_{0} }}^{2} }} + \frac{{\sigma_{{ac_{y} }}^{2} }}{{\sigma_{{y_{0} }}^{2} }}} }}. $$

**Equation 78:**

78 $$ A^{m} = \frac{1}{{\sqrt {1 + \left( {1 + \frac{{\mu_{{x_{0} }}^{2} }}{{\sigma_{{x_{0} }}^{2} }}} \right)\left( {\frac{{\sigma_{{mu_{x} }}^{2} }}{{\sigma_{{x_{0} }}^{2} }} + \frac{{\sigma_{{mc_{x} }}^{2} }}{{\sigma_{{x_{0} }}^{2} }}} \right)} \sqrt {1 + \left( {1 + \frac{{\mu_{{y_{0} }}^{2} }}{{\sigma_{{y_{0} }}^{2} }}} \right)\left( {\frac{{\sigma_{{mu_{y} }}^{2} }}{{\sigma_{{y_{0} }}^{2} }} + \frac{{\sigma_{{mc_{y} }}^{2} }}{{\sigma_{{y_{0} }}^{2} }}} \right)} }}. $$

should read:

78 $$ A^{m} = \frac{1}{{\sqrt {1 + \left( {1 + \frac{{\mu_{{x_{0} }}^{2} }}{{\sigma_{{x_{0} }}^{2} }}} \right)\left( {\sigma_{{mu_{x} }}^{2} + \sigma_{{mc_{x} }}^{2} } \right)} \times \sqrt {1 + \left( {1 + \frac{{\mu_{{y_{0} }}^{2} }}{{\sigma_{{y_{0} }}^{2} }}} \right)\left( {\sigma_{{mu_{y} }}^{2} + \sigma_{{mc_{y} }}^{2} } \right)} }} $$

In addition, in the Material and Methods section, under the subheading ‘Software’, the link where the data are available has been changed, from ‘systemsbiology.nl under the SOFTWARE tab’ to: https://github.com/esaccenti/CorrelationCorruption.

